# Intramuscular Injection of Bone Marrow Stem Cells in Amyotrophic Lateral Sclerosis Patients: A Randomized Clinical Trial

**DOI:** 10.3389/fnins.2020.00195

**Published:** 2020-03-24

**Authors:** Emilio Geijo-Barrientos, Carlos Pastore-Olmedo, Pedro De Mingo, Miguel Blanquer, Joaquín Gómez Espuch, Francisca Iniesta, Natalia García Iniesta, Ana García-Hernández, Carlos Martín-Estefanía, Laura Barrios, José M. Moraleda, Salvador Martínez

**Affiliations:** ^1^Institute of Neurosciences, Universidad Miguel Hernández-CSIC, Alicante, Spain; ^2^Clinical Neurophysiology Service, San Juan University Hospital, Alicante, Spain; ^3^Service of Clinical Neurophysiology, Virgen de la Arrixaca University Clinical Hospital, Murcia, Spain; ^4^Hematopoietic Stem Cell Transplant and Cell Therapy Unit, Hematology Service, Virgen de la Arrixaca University Hospital, University of Murcia, Murcia, Spain; ^5^Institute for Bio-Health Research of Murcia (IMIB-Arrixaca), Murcia, Spain; ^6^Department of Applied Statistics, SGAI-CSIC, Madrid, Spain

**Keywords:** ALS, MUNE, MUNIX, CMAP scan, D50, fiber density, motor units

## Abstract

**Background:**

Preclinical studies suggest that stem cells may be a valuable therapeutic tool in amyotrophic lateral sclerosis (ALS). As it has been demonstrated that there are molecular changes at the end-plate during the early stages of motorneuron degeneration in animal models, we hypothesize that the local effect of this stem cell delivery method could slow the progressive loss of motor units (MUs) in ALS patients.

**Methods:**

We designed a Phase I/II clinical trial to study the safety of intramuscularly implanting autologous bone marrow mononuclear cells (BMMCs), including stem cells, in ALS patients and their possible effects on the MU of the tibialis anterior (TA) muscle. Twenty-two patients participated in a randomized, double-blind, placebo-controlled trial that consisted of a baseline visit followed by one intramuscular injection of BMNCs, follow-up visits at 30, 90, 180, and 360 days, and an additional year of clinical follow-up. In each patient, one TA muscle was injected with a single dose of BMMCs while the contralateral muscle was given a placebo; the sides were selected randomly. All visits included a complete EMG study of both TA muscles.

**Results:**

Our results show that (1) the intramuscular injection of BMMCs is a safe procedure; (2) ALS patients show heterogeneities in the degree of TA injury; (3) a comparison of placebo-injected muscles with BMMC-injected muscles showed significant differences in only one parameter, the D50 index used to quantify the Compound Muscle Action Potential (CMAP) scan curve. This parameter was higher in the BMMC-injected TA muscle at both 90 days (placebo side: 29.55 ± 2.89, *n* = 20; experimental side: 39.25 ± 3.21, *n* = 20; *p* < 0.01) and 180 days (placebo side: 29.35 ± 3.29, *n* = 17; experimental side: 41.24 ± 3.34, *n* = 17; *p* < 0.01).

**Conclusion:**

This procedure had no effect on the TA muscle MU properties, with the exception of the D50 index. Finding differences in just this index supports the fact that it may be much more sensitive than other electrophysiological parameters when studying treatment effects. Given the low number of patients and their heterogeneity, these results justify exploring the efficacy of this procedure in further patients and other muscles, through Phase II trials.

**Clinical Trial Registration:**

www.clinicaltrials.gov (identifier NCT02286011); EudraCT number 2011-004801-25.

## Introduction

Amyotrophic lateral sclerosis (ALS) is a neurodegenerative disorder with no curative treatment that is characterized by progressive muscle weakness and a fatal prognosis. It shows marked phenotypic heterogeneity with very varied clinical presentation (Al-Chalabi et al., 2016). Familial ALS has been related to a number of genetic mutations, as have some cases of sporadic ALS (Turner et al., 2013; Gibson et al., 2017). Although ALS is almost certainly a systemic that involves some decreased functional of the upper and lower motor neurons, most therapeutic efforts are aimed primarily at prolonging or improving survival by stopping the progressive loss of motor units (MUs).

A promising therapeutic strategy for ALS, developed in recent years, involves the use of cell therapy techniques based on stem cell transplantation. Mesenchymal stem cells (MSCs) have been used in animal models of ALS as well as ALS patients, although through different techniques and delivery routes. Preclinical studies of animal models of motor neuron diseases demonstrate the efficacy of MSCs in targeting pathogenic mechanisms and slowing the progression of the disease (Suzuki et al., 2008; Pastor et al., 2013; Rando et al., 2018). Phase I/II clinical trials on patients have demonstrated the safety of both neural stem cells and MSC, delivered using different routes, but have not yet shown definitive evidence of efficacy in ALS patients (Blanquer et al., 2012; Faravelli et al., 2014; Thomsen et al., 2014; Petrou et al., 2016); many authors consider that this would require well-designed and robust randomized clinical trials (reviewed in Mazzini et al., 2018). Currently, several research groups are investigating the use of stem cells applied in various ways to ALS patients, trying to demonstrate an inhibition of the disease progression (Scott, 2017). To date, Phase I/II clinical trials have been carried out involving intrathecal injections of autologous adipose-derived mesenchymal stromal cells (Staff et al., 2016), and intrathecal injections of bone marrow mesenchymal stromal cells (Syková et al., 2017). Bone-marrow-derived MSCs have been used in ALS patients by intraspinal injection (Blanquer et al., 2012) or by intrathecal injection after their induction to secrete neurotrophic factors (Petrou et al., 2016). These stem cells could have protective actions on motoneurons mediated by several mechanisms, including the secretion of cytokines (with anti-inflammatory effect) and/or neurotrophic factors (Sadan et al., 2009).

During the progression of ALS there are early pathological changes affecting the motor neuron axon terminals and the neuromuscular junction; these changes precede motor neuron degeneration and the onset of symptoms, which suggest that degeneration of motor neurons would be initiated in the periphery by this distal axonopaythy and would progress centrally following a “dying back” pattern (Coleman and Perry, 2002; Fischer et al., 2004; Dupuis and Loeffler, 2007; Dadon-Nachum et al., 2010; Moloney et al., 2014). This “dying back” hypothesis is supported (Fischer et al., 2004) by findings in the SOD1^G93A^ mouse model (where denervation of neuromuscular synapses and ventral root axon loss precedes the loss of motor neuron cell bodies) and in ALS patients (where denervation and innervation changes were detected in peripheral muscles before the detection of pathological changes in the motor neurons). Importantly, a decreased level of synaptic protein in the neuromuscular junctions of limb muscles in ALS patients has recently been found (Liu et al., 2013). The presence of early neuromuscular junction damage and distal axonopathy suggests that a possible therapeutic approach in ALS is to act on the axon terminal and/or the neuromuscular junction for protecting the motor neurons from degeneration initiated in the periphery. The direct intramuscular implantation of stem cells could be a way to accomplish this goal because these cells release neurotrophic factors and cytokines that could act locally on the axon terminals or neuromuscular junctions due to their trophic support and anti-inflammatory actions (Sadan et al., 2009). In fact, our group has shown that the intramuscular injection of bone-marrow-derived mesenchymal cells in an animal model of motor neuron degeneration causes an increase in the size of the end-plates correlating with increased motor neuron survival (Pastor et al., 2013). In addition, neurotrophic factors released locally are transported to the motoneuron soma by retrograde transport (Suzuki et al., 2008; Pastor et al., 2013), where they may promote cell survival. On these grounds, the direct intramuscular implantation of bone-marrow-derived MSCs is an alternative way to deliver these cells to ALS patients. The intramuscular implantation of stem cells has been tested only in one clinical trial involving a very limited number of patients with ALS and using autologous bone-marrow-derived stem cells modified to enhance their secretion of neurotrophic factors (GDNF; Petrou et al., 2016).

We have carried out a clinical trial to study the safety and possible effects of the intramuscular implantation of autologous bone-marrow-derived stem cells on the functional properties of the MUs of a muscle of ALS patients (the tibialis anterior, TA) during the progression of the disease. The trial design was based on the unilateral injection of stem cells with the contralateral muscle used as a control. This approach has recently been confirmed as a valid strategy in clinical trials on ALS (Rushton et al., 2017).

## Materials and Methods

A Phase I/II clinical trial was designed to study the safety of implanting intramuscularly autologous bone marrow mononuclear cells (BMMCs, including stem cells). We also studied a complete set of EMG parameters to determine the possible effects of intramuscular BMMCs on the functional properties of MUs during ALS progression. The study was conducted in accordance with the trial protocol and all applicable regulatory requirements. The trial protocol was approved by the Clinical Research Ethics Committees of the participating institutions and was registered at www.clinicaltrials.gov (identifier NCT02286011) as well as in the European Clinical Trials Database (EudraCT number 2011-004801-25). All patients provided written informed consent before participating in the study.

We selected the TA muscle because it is a well-studied muscle in both normal subjects and ALS patients (Trojaborg et al., 2002; Neuwirth et al., 2011), is easily accessible, and allows detailed EMG studies; its size and volume also permits adequate recovery of tissue after the intramuscular injection of a minimal volume (2 ml) of solution with cells or placebo. The TA muscle is innervated by the deep peroneal nerve. The TA muscle is systematically studied in ALS patients and damage to it causes significant deficiencies in the mobility of the lower limb; all this makes the TA a very important muscle when evaluating the progression of ALS in respect to the capacity to walk properly.

### Patients and Trial Protocol

The main inclusion criteria were a diagnosis of probable or definite ALS according to the revised El Escorial criteria established by the World Federation of Neurology and a Medical Research Council (MRC) score of 4 or 5 on dorsiflexion of both feet. The main exclusion criteria were diabetes mellitus, diseases that may occur with polyneuropathies, and previous pathology of the peripheral nervous system that affects one or both lower limbs with or without clinically evident neurological sequelae. Twenty-two patients were recruited and all of them were diagnosed of sporadic ALS. Each of whom fulfilled all the inclusion criteria and none of the exclusion criteria; this sample is similar in size to those used in Phase I clinical trial on ALS using transplanted stem cells (e.g., Mazzini et al., 2015; Petrou et al., 2016). The complete trial protocol may be obtained from the corresponding author.

Once included in the trial, the patients underwent a basal visit (day 0) that included a study of the anesthetic risk, a neurological exploration, and an EMG study of both TA muscles. The day after the basal visit (day 1), bone marrow was harvested under sedation, and BMMCs were obtained and intramuscularly injected by the hematology team. In each patient, one TA was injected with BMMCs (experimental side) while the contralateral muscle received normal saline (control side); the control and experimental sides were determined randomly (see below: randomization procedure). Post-injection there were follow-up visits at days 30, 90, 180, and 360. Identical clinical and electrophysiological assessments were performed at the basal visit and at the follow-up visits. In addition, a phone evaluation was conducted on days 7, 60, 120, and 270, as well as quarterly during the second year of follow-up. Safety was established primarily as the rate of serious and non-serious procedure-related adverse events (AEs), as defined by the CONSORT group (Ioannidis et al., 2004). AEs were recorded whenever they occurred.

### Randomization Procedure

The lower limbs of the 22 patients enrolled in the study were randomly allocated in a 1:1 ratio to receive either BMMC or placebo in the TA muscle. The experimental and control sides were assigned randomly (by an independent statistician). The assignation of experimental or control side was known only by the hematologists who administered the treatment and the infusion was their only contact with the patients. Both the patients and the physicians that performed the clinical and electrophysiology examinations were blinded to the assigned treatment until the end of study. The procedure was exactly the same in both lower limbs (the injected volumes of BMMCs and placebo were the same; in addition, and both were injected in equivalent locations with a similar needle).

### Cell Injection Procedure

Autologous bone marrow (120 ml) was harvested, under sedation with Propofol (0.5 mg/kg) and Fentanyl (50–100 μg), through multiple aspirations from the posterior iliac crests. Clinical-grade BMMCs were manufactured under best manufacturing practice–compliant conditions for clinical research products (IP: 06-074) in the clean room facility at Virgen de la Arrixaca University Clinic Hospital (Murcia, Spain). The BMMCs and placebo were both manufactured and provided by the Cell Production Unit of the Virgen de la Arrixaca University Clinical Hospital according to the randomization sequence. A ficoll density gradient separation was performed to isolate the mononuclear fraction. The mononuclear cells were then re-suspended in 2.1 ml of normal saline and loaded into two 1-ml syringes. The remaining 0.1 ml was used to assess the number of mononuclear cells obtained; the viability of the cells was measured using trypan blue exclusion, and the numbers of CD34 + and CD133 + cells were determined through flow cytometry. A Yaşargil arm equipped with a microinjector and a controlled infusion device was used to infuse the cells. Four 0.5-ml infusions were performed around the recording and injection point (see below). A median of 499 × 10^6^ (range 206–1086 × 10^6^) BMMCs were infused. The same procedure was performed in the contralateral TA but with normal saline. The patient was blinded throughout the infusion process, and the hematology team took no part in evaluating the patients.

### TA Muscle Strength Measurements

The muscle strength (MS) of the TA muscles was measured at all visits using the MRC scale in both legs of each patient prior to the EMG study.

### Electromyography

Both TA muscles of each patient were studied at all visits using a Natus Neurology Incorporated (Middleton, WI, United States) electromyography apparatus running either Synergy (Version 22.0.0.144) or Viking (EDX Version 20.1.30 CareFusion) EMG software. During the recordings, the skin temperature was controlled and maintained between 34 and 36°C using an infrared lamp whenever necessary. The same person (P. de M.) generated all the EMG records and took all the MS measurements in all the patients and at all the visits.

To evaluate the functional status of the TA muscles, we used the MS in addition to the following electrophysiological parameters: compound muscle action potential (CMAP) peak amplitude and area, fiber density (FD), CMAP scan stimulus–amplitude curve and D50 index, single motor unit potential (SMUP) and motor unit number estimation (MUNE quantified using statistical technique), and indices of motor unit number (MUNIX) and motor unit size (MUSIX). We used multiple EMG parameters to search for consistency among the parameters quantifying the various MU properties.

#### Recording and Injection Point CMAP Recordings

**FIGURE 1:**
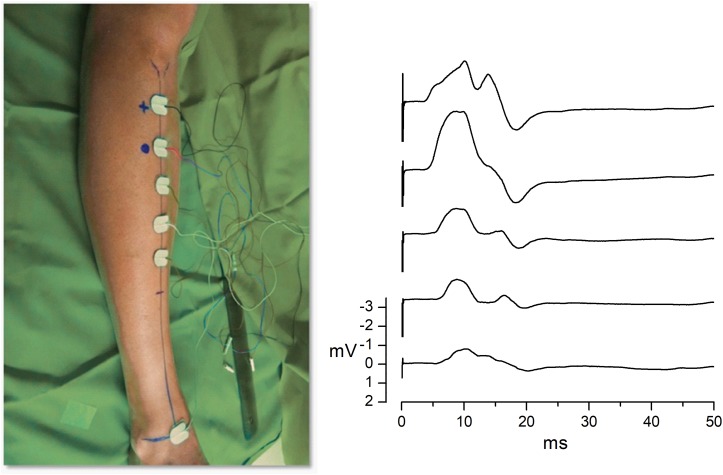
Determining the recording and injection point. CMAP recordings. To determine the recording and injection point for each TA muscle, we mapped the CMAP with five surface recording electrodes placed on the surface of the muscle. We always used the same type of surface electrodes (Ambu Neuroline 700 Single Patient Surface Adhesive Electrodes; Ref. 70010-k/C/12). The left panel illustrates the position of the five active electrodes, as well as the reference electrode, on the right leg of one patient. The five recording electrodes were placed using the same bone references in all patients: 1 cm lateral to the tibial crest on the line linking the tibial tuberosity and the midpoint of the bi-malleolar line (blue lines in the left panel). The distance between the tibial crest and the most proximal electrode, as well as the distance between the successive four electrodes was 10% of the distance between the tibial tuberosity and the bi-malleolar line. A bipolar stimulation skin electrode (Medelec; Ref. FT 296.180 TP, not shown in the picture) was placed on the fibular neck. The ground electrode (Ambu Neuroline Ground Neurology Surface, not shown in the picture) was placed between the stimulus electrode and the uppermost electrode. The right panel shows the CMAP recordings elicited by supramaximal stimulation and recorded simultaneously by the five recording electrodes (3–3000 Hz bandpass filter, without a 50-Hz notch filter). Note the progressive increment of the distal latency of CMAP and different amplitudes in each channel. The recording point was determined as the position of the electrode where the maximum amplitude of CMAP was recorded with minimal contamination from other muscles (electrode no. 2 in this case). This position was marked with indelible ink so it could be used as a reference for the intramuscular injections and recordings in successive visits. Calibration: 50 ms/5 mV dot intervals. The CMAP was recorded using standard motor nerve conduction techniques at the basal and follow-up visits. After a supramaximal superficial stimulation of the peroneal nerve in the fibula neck, three or four artifact-free recordings were superimposed to obtain the best response.

The CMAP peak amplitude and area were measured using standard motor nerve conduction techniques in the basal and follow-up visits. Given that the size of the CMAP recorded with surface electrodes depends on the size of the electrode and its position with respect to the muscle (van Dijk et al., 1995), we designed a method to minimize variability in CMAP from one visit to another that was independent of the eventual treatment or placebo. At the basal visit (day 0), we chose a recording and injection point (RP) on each TA muscle; the RP was the point at which the CMAP had its maximum amplitude and minimal contamination (by noise or volume conducted signals), after mapping the entire muscle of each patient (Figure 1). This RP was maintained in the follow-up visits and was used for all EMG measurements. Also, the RP was used as a reference for the intramuscular injections of BMMCs and the placebo.

#### Fiber Density

We measured FD according to the original description (Stålberg et al., 1975) using Synergy Software. FD has been used extensively in a number of neurogenic disorders, including ALS (Cruz-Martínez et al., 1983; Liu et al., 2016). Currently, there is a broad consensus that FD is a measurement of the MU size, since it measures the local concentration of muscle fibers belonging to a single MU.

#### CMAP Scan Curve and D50 Index

**FIGURE 2:**
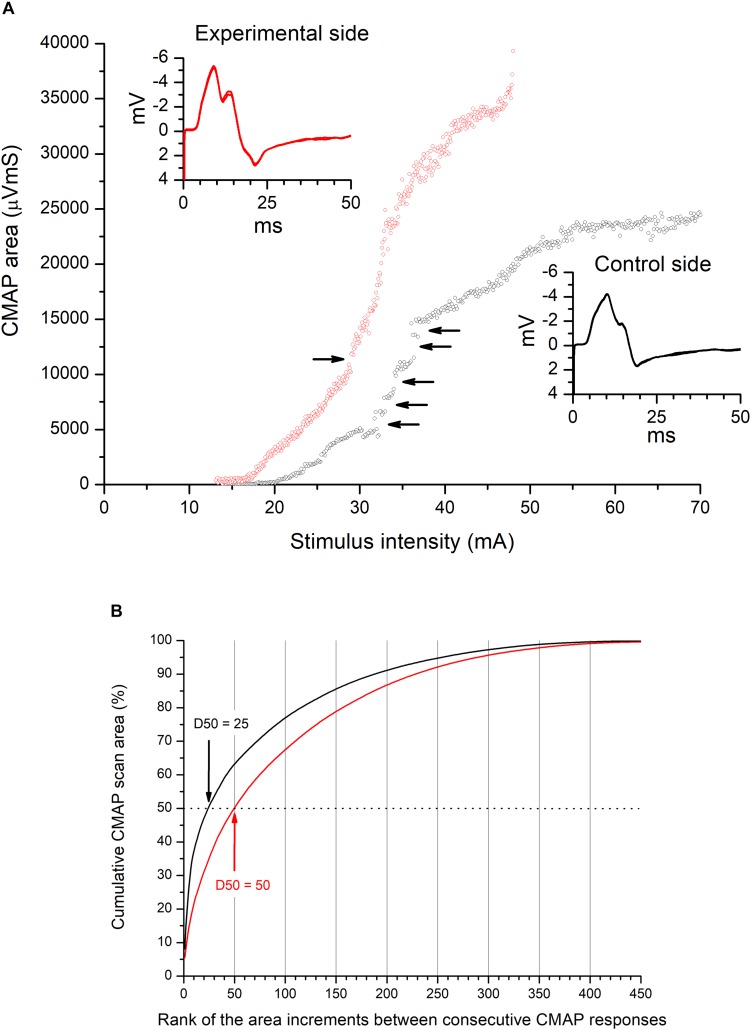
CMAP scan stimulus–amplitude recordings and D50 index. **(A)** The CMAP scan stimulus–amplitude curve shows the relationship between CMAP size and stimulus intensity, plotted across the range of stimulus intensities from the threshold (S_0_) to the supramaximal intensity (S_100_). Each plot shows 500 consecutive values of the CMAP area (mVms) plotted against their stimulus intensities (mA) (0.1 ms duration; delivered at 2 Hz frequency), equally distributed between S_0_ and S_100_ in a downward direction. Recordings were made on the control (black symbols) and experimental (red symbols) TA muscles of patient #17 at the + 30-day visit. The comparison of the two curves shows that there are many more CMAP scan discontinuities (marked by horizontal arrows) in the control TA muscle, i.e., more numerous, larger steps during the CMAP scan of the sequential recruiting of the whole muscle fiber population. These discontinuities are caused by a decrease in MU number and an increase in MU size, and may be quantified by the D50 index. The inset shows the shape of the maximum CMAP recorded on both sides. **(B)** Calculation of the D50 parameter. Each line (control side black, experimental side red) shows the cumulative sum of the increments in the CMAP area between successive responses after ranking these increments from largest to smallest; the D50 parameter is the number of increments necessary to reach 50% of the total sum. For the experimental side (left leg), D50 = 50; for the control side (right leg), D50 = 25. This increase in D50 index on the experimental side with respect to the control side was associated with an increase in axon excitability (measured as the maximum slope of the CMAP scan curve: 0.24 mV/mA on the experimental side and 0.07 mV/mA on the control side).

The CMAP scan curve is the relationship between the stimulus strength and the size of the CMAP (amplitude or area) obtained across the whole range of stimulus intensities, from threshold to maximum response (Visser and Blok, 2009); this curve characterizes the sequential recruitment of motor axons with different thresholds. We constructed this curve from CMAP recordings obtained at the RP (Figure 2A). To quantify the CMAP scan curve and its discontinuities, we used the D50 index (Sleutjes et al., 2014). Like other indices proposed to quantify the CMAP scan curve (Maathius et al., 2013; Bostock, 2016), the D50 index is a single number that quantifies the number and size of the steps between consecutive responses during the sequential recruitment of the entire population of motor axons innervating a given muscle. The D50 index provides information on the number and size of the functional MUs. To determine D50 (Figure 2B), the successive steps of the CMAP scan curve were ordered according to their size, starting from the largest; the D50 index is the minimum number of steps whose sum is the 50% of the maximum CMAP area; this algorithm has been described previously (Sleutjes et al., 2014). For this purpose, we used an *ad hoc* template.

#### Single MU Potential Size and Statistic MU Number Estimation

The MUNE was calculated as the quotient between the maximum CMAP area and the average size of the SMUP areas recorded using the same electrode. To measure the SMUP, we used the statistical method in Daube (2006). We found a number of practical technical limitations, particularly when the CMAP areas were small or very small: measuring small SMUPs (area < 25 μVms or peak amplitude < 10 μV) results in spuriously high MUNE values, since their small size has a disproportionate impact on the MUNE calculation (Bromberg, 2006). We only accepted SMUP areas larger than 25 μVms as valid values. This rejection of MUNE and SMUP measurements was particularly marked in the later visits of patients with severely affected TA muscles; this resulted in a very low number of MUNE and SMUP measurements in these cases.

#### Indices of MU Number and MU Size

The MUNIX was calculated using an algorithm previously described (Nandedkar et al., 2004). MUNIX was calculated using an *ad hoc* program written by EG-B and CP-O in an Excel^®^ environment. Calculating the MUNIX also provides the MU size index (MUSIX), measured in μV, and obtained by dividing the amplitude of the CMAP by the MUNIX. The MUSIX may be proportional to the size of the MUs and in some sense may reflect SMUP amplitude. Both parameters are useful for studying MU loss and size during the progression of ALS patients and, for this reason, were also selected for this clinical trial (Nandedkar et al., 2010; Neuwirth et al., 2010).

### Statistical Methods

All the data are presented as the mean ± SEM and the number of cases. All comparisons were made using non-parametric tests (Mann–Whitney rank sum test for independent samples and Wilcoxon signed rank test for paired samples). These comparisons were made using SigmaStat v3.5 (Systat software Inc., San Jose, CA, United States). To quantify the progression of a parameter in time, we compared the values from the basal visit with the values at the 180- or 360-day visits using the Wilcoxon signed rank test for paired samples for those patients who continued in the study far as these visits. For all parameters, we used a generalized estimating equations model to explain the effects of treatment with BMMCs in ALS progression. Muscle heterogeneity was analyzed using hierarchical cluster analysis. The generalized estimating equations model and cluster analysis were carried out with IBM SPSS Statistics v24 software. A *p* value of < 0.05 was considered significant.

## Results

**FIGURE 3:**
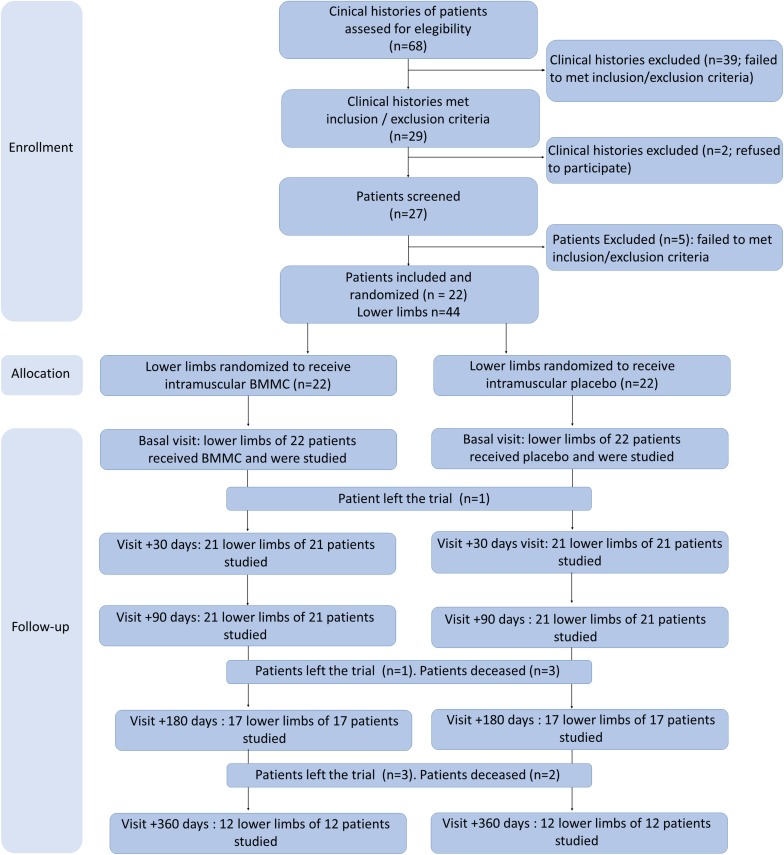
Flowchart of the study. It was impossible to complete the EMG study in some patients, or some data could not be recovered: for this reason, the number of cases shown in the figures may not be the same as the number of patients examined at each visit.

A total of 22 patients, recruited from November 2014 to December 2015, were studied. The demographic and clinical characteristics of these patients are presented in Table 1 and the flow diagram of the study is illustrated in Figure 3. The autologous transplantation of BMMCs (including CD34 + stem cells) into the TA muscle was a safe procedure. No serious AEs related to the procedure were detected (Table 2); a complete description of all AEs recorded during the trial is given in Table 3. The echography of the TA muscles showed no signs of local inflammation or other lesions at the injection site; this also indicates that it is unlikely the intramuscular injection interfered with the subsequent EMG studies.

**TABLE 1:** Demographic data and classification of patients.

Patient #	Age range (years)	Time of clinical evolution (months)	Last visit (days)	Disease start	ALS diagnosis
1	47–51	119	360	Spinal	Definite
2	67–71	41	90	Bulbar	Probable
3	52–56	88	360	Spinal	Definite
4	52–56	53	360	Bulbar	Definite
5	47–51	5	360	Spinal	Probable
6	62–66	9	90	Bulbar	Definite
7	32–36	17	180	Spinal	Definite
8	57–61	16	90	Spinal	Definite
9	67–71	20	90	Bulbar	Definite
10	37–41	57	360	Spinal	Definite
11	57–61	17	180	Spinal	Definite
12	57–61	34	360	Spinal	Definite
13	52–56	11	360	Spinal	Definite
14	52–56	28	360	Spinal	Probable
15	52–56	12	180	Spinal	Definite
16	57–61	13	360	Spinal	Definite
17	67–71	7	180	Spinal	Definite
18	47–51	54	360	Spinal	Definite
19^a^	67–71	42	180	Bulbar	Definite
20	42–46	12	360	Spinal	Definite
21	62–66	12	Basal	Spinal	Definite
22	42–46	43	360	Spinal	Definite
Mean ± SEM	55.6 ± 2.1	32.3 ± 6.1			
Range	33–70	5–119			

*Gender: 17 males, 5 females. Patients numbered according to the recruitment date. ^*a*^This patient did not attend the +90-day visit.*

**TABLE 2:** Serious adverse events recorded in the clinical trial.

Patient#	Category	Description	Onset day	Stop day	Duration	Procedure relationship	Unexpected
2	Hospitalization	Respiratory insufficiency	+375	+395	1	Not related	No
2	Death	Cardiorespiratory arrest	+395	+395	21	Not related	No
4	Death	Cardiorespiratory arrest	+413	+413	1	Not related	No
6	Hospitalization	Pneumonia	+88	+89	2	Not related	No
6	Hospitalization	Pneumonia	+108	+113	6	Not related	No
6	Death	Cardiorespiratory arrest	+175	+175	1	Not related	No
8	Death	Cardiorespiratory arrest	+115	+115	1	Not related	No
9	Hospitalization	Respiratory tract infection	+142	+145	4	Not related	No
9	Death	Cardiorespiratory arrest	+145	+145	1	Not related	No
11	Death	Cardiorespiratory arrest	+334	+334	1	Not related	No
12	Hospitalization	Herniorrhaphy	+268	+269	2	Not related	No
12	Hospitalization	Urinary tract infection	+280	+292	13	Not related	No
15	Death	Cardiorespiratory arrest	+227	+227	1	Not related	No
17	Death	Cardiorespiratory arrest	+318	+318	1	Not related	No
19	Hospitalization	Respiratory insufficiency	+56	+63	8	Not related	No

*Fifteen serious adverse events (SAEs) were recorded. None were associated with the intramuscular administration of BMMCs. There were 7 hospitalizations and 8 deaths, all of which were considered unrelated to the procedure. At 24 months, there had been 15 SAEs in 10 patients (47.6%): 7 hospitalizations in 5 patients (23.8%) and 8 deaths (38.0%). No protocol-defined withdrawal criteria (tumors, hemorrhages at the point of injection or ischemia) were reported. The primary endpoint of the study was achieved because none of the 21 patients treated (with AE follow-up) experienced any serious procedure-related AE and, indeed, all the non-severe AEs related to the procedure were mild (grade ≤ 2), and all were transient.*

**TABLE 3:** Adverse events recorded in the clinical trial.

Number of patients treated with AE follow-up	21
Number of non-serious adverse events	29
Non-serious adverse events per patient (mean/median/range)	1.8/1/1–5
Number of non-serious, not-procedure related events	22
Non-serious, non-procedure related events per patient (mean/median/range)	1.4/1/0–4
Number of non-serious, procedure related events	7
Non-serious, procedure related events per patient (mean/median/range)	0.4/0/0–2
Number of serious adverse events	15
Serious adverse events per patient (mean/median/range)	1.5/1/1–3
Number of non-serious, non-procedure related events	15
Serious, non-procedure related events per patient (mean/median/range)	1.5/1/1–3
Number of serious, procedure related events	0
Serious, procedure related events per patient (mean/median/range)	ND
Patients with **>** 1 non-serious adverse event	16
Patients with **>** 1 non-serious procedure-related adverse event	6
Patients with **>** 1 serious adverse event	10
Patients with **>** 1 serious procedure-related adverse event	0

*The table summarizes the safety data based on the frequency of adverse events (AEs) and serious adverse events (SAEs). Forty-four AEs were recorded. Fifteen (34%) AEs were rated as serious. Overall, 16 patients (76.2%) suffered non-serious AEs (10 non-procedure-related and 6 procedure-related), and 10 patients (47.6%) experienced SAEs, none of which were procedure-related. Six patients reported 7 non-serious procedure-related AEs, consisting of 2–3 days of CTCAE (version 4.0) grade 1–2 post-bone marrow harvest pain (except one patient, who experienced some degree of pain for 29 days) and one episode of dizziness. There were no significant changes in any laboratory parameters, including blood counts, biochemistry, renal and hepatic function, and thyroid function. Ultrasound images of the anterior tibialis muscle on day + 7 revealed no significant pathological change at the injection site (neither infection nor tumor formation) in any patient. No expected adverse reactions (local erythema, pain at the injection site, allergic reactions and local infections) were identified.*

**FIGURE 4:**
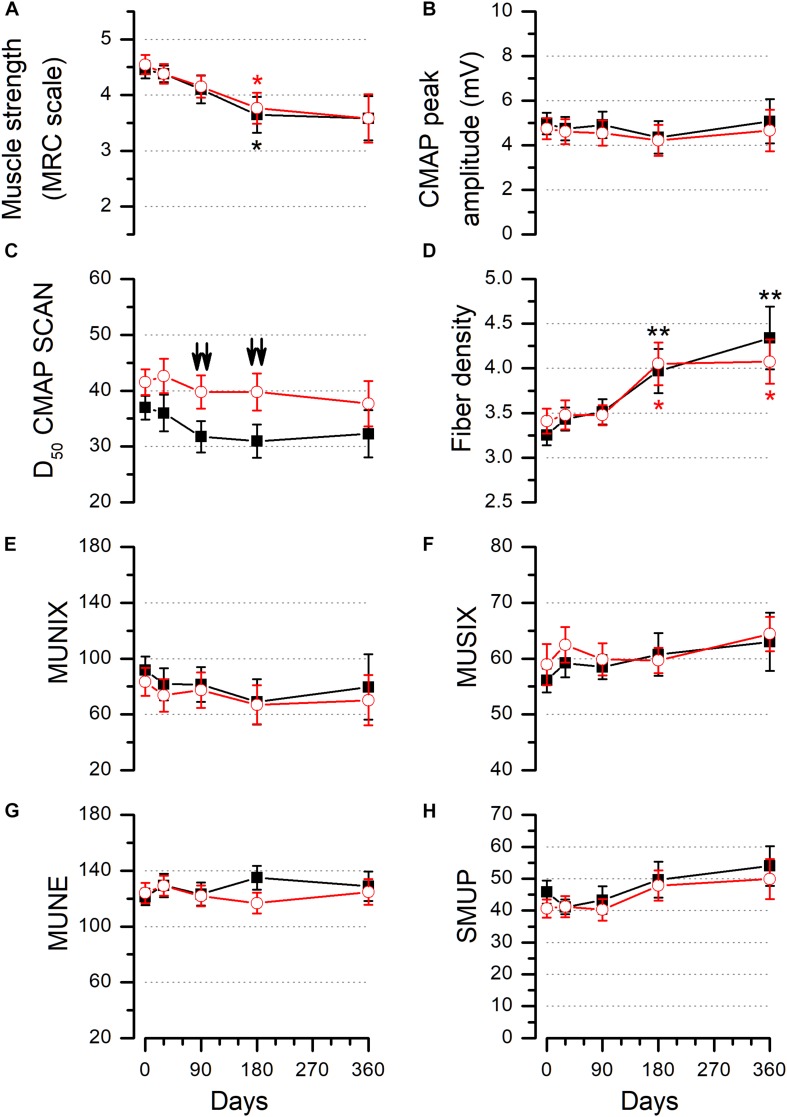
Muscle strength and electrophysiological parameters measured in control and experimental TA muscles. Values of MS **(A)**, CMAP peak amplitude **(B)**, D50 **(C)**, FD **(D)**, MUNIX **(E)**, MUSIX **(F)**, statistical MUNE **(G)**, and SMUP **(H)** measured in the TA muscles of all patients studied. Asterisks in panels **(A)** and **(D)** show the presence of statistically significant differences between the basal visit and the 180- or 360-day visit on the control (black asterisks) or on the experimental (red asterisks) side. Comparisons with the Wilcoxon signed rank test for paired samples. Downward arrows (↓) in panel **(C)** show differences between the control and experimental sides at the 90- and 180-day follow-up visits (Wilcoxon signed rank test for paired samples). In panels **(A)**, **(C)**, and **(D):** single symbol: *p* < 0.05, two symbols: *p* < 0.01, three symbols: *p* < 0.001. Numbers of measurements for each parameter in the successive visits are as follows: MS (control and experimental sides): 20, 18, 19, 17, 12; CMAP control side: 19, 20, 20, 17, 12 and experimental side: 20, 20, 20, 17, 12; D50 (control and experimental sides): 22, 21, 20, 17, 12; FD (control and experimental sides): 22, 21, 20, 15, 12; MUNIX (control and experimental sides): 19, 19, 19, 16, 11; MUSIX (control and experimental sides): 20, 20, 20, 17, 12; MUNE control side: 19, 19, 17, 12, 8, and experimental side: 15, 17, 17, 12, 9; SMUP control side: 19, 17, 17, 9, 8, and experimental side: 17, 16, 16, 12, 9.

Figure 4 shows the average values of all functional parameters measured on the control and experimental sides. We compared the control and experimental muscles using a generalized estimating equations model (see section “Materials and Methods”); this test showed no correlation with the treatment in any of the parameters studied. However, when comparing the values of the control and experimental sides at individual visits, we did find differences in the D50 index. This parameter was grater in the BMMC-injected TA muscle with respect to the control side (Figure 4C) at 90 days (placebo side: 29.55 ± 2.89, *n* = 20; experimental side: 39.25 ± 3.21, *n* = 20) and 180 days (placebo side: 29.35 ± 3.29, *n* = 17; experimental side: 41.24 ± 3.34, *n* = 17). These differences between the two sides were statistically significant when compared either using the Wilcoxon signed rank test for paired samples (*p* < 0.01 at 90 and 180 days) or the Mann–Whitney rank sum test for independent samples (*p* < 0.05 at 90 and 180 days). In addition to these differences, we noted that FD and MS changed between the different study visits (Figures 4A,D). FD increased significantly from the basal visit to the 180-day visit (control side: 3.18 ± 0.53, 3.97 ± 0.25, *n* = 15, *p* = 0.005; experimental side: 3.37 ± 0.19, 4.05 ± 0.24, *n* = 15, *p* = 0.041) and the 360-day visit (control side: 3.17 ± 0.17, 4.27 ± 0.38, *n* = 11, *p* = 0.002; experimental side: 3.48 ± 0.24, 4.06 ± 0.27, *n* = 11, *p* = 0.042) (Figure 4D). MS decreased, diminishing significantly progression from the basal visit to the 180-day visit on both sides (control side: 4.35 ± 0.19, 3.65 ± 0.32, *n* = 17, *p* = 0.037; experimental side: 4.47 ± 0.21, 3.77 ± 0.28, *n* = 17, *p* = 0.014) (Figure 4A; see section “Materials and Methods” section for a description of how the progression of various parameters was assessed during the study).

**FIGURE 5:**
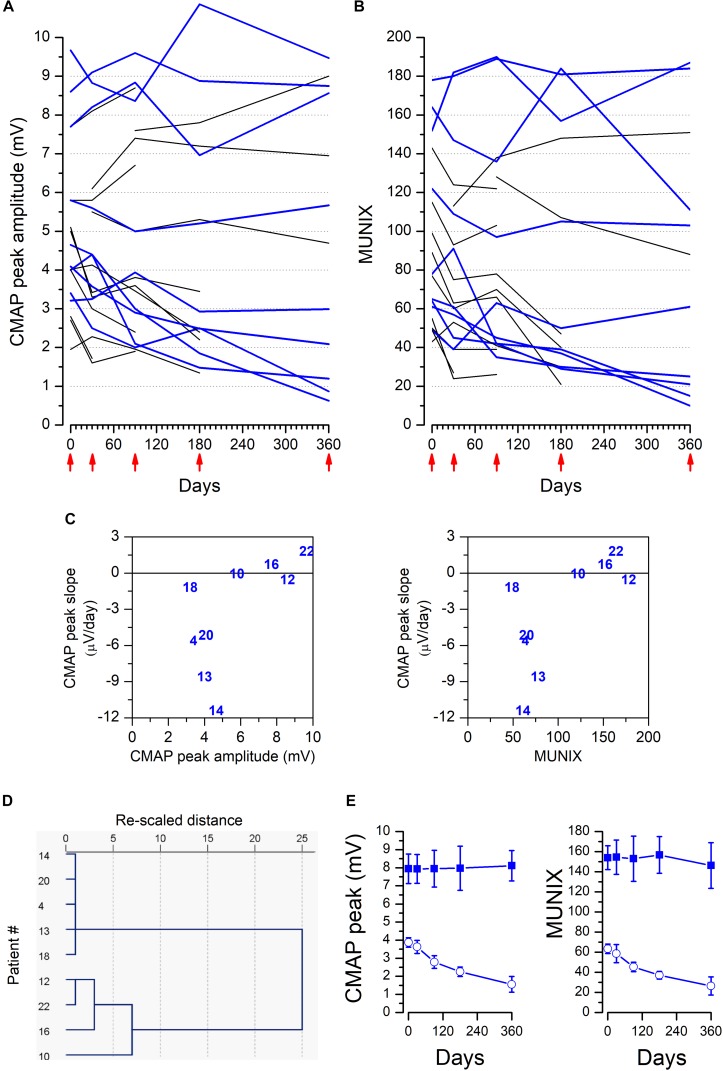
Heterogeneity of TA muscles in ALS patients. Values of CMAP peak amplitude **(A)** and MUNIX **(B)** measured on the control side for all patients studied. Blue lines represent patients who completed the study from the basal to the 360-day visit; either the remaining patients did not finish the study (due to death or abandonment) or the data from a visit could not be recovered. The red arrowheads point to the time of the five visits (basal, 30, 90, 180, and 360 days). **(C)** Correlation between the slope of the CMAP peak amplitude measured on the control side in the nine patients that completed the study and the CMAP peak amplitude (left) or MUNIX (right) measured on the control side at the basal visit. The slope of the peak CMAP amplitude was taken from the linear regression of the CMAP values measured on the control side at all five visits. **(D)** Dendrogram produced by hierarchical cluster analysis using CMAP, MUNIX, D50, and FD at the basal visit and the slope of the CMAP measured on the control side. **(E)** Mean ± SEM of the CMAP peak amplitude and MUNIX values of patients #10, #12, #16, and #22 (filled symbols) and patients #4, #13, #14, #18, and #20 (open symbols).

We observed a high degree of heterogeneity among TA muscles of different patients when considering the individual CMAP amplitude and MUNIX values measured on the control side (Figures 5A,B); some had high CMAP peak amplitude or MUNIX values at the basal visit, and these values remained fairly stable throughout the study; in contrast, other patients showed lower values that fell further during the study. We undertook a hierarchical cluster analysis using the values of CMAP peak amplitude, FD, D50, and MUNIX measured on the control side at the basal visit, and a further parameter to estimate the evolution during the follow-up period (the slope of the linear fit of the CMAP peak on the control side at the five visits). The cluster analysis was run only for the 9 patients who completed all visits (blue lines in Figures 5A,B). Figures 5C,D show the correlation between the slope of the CMAP peak and CMAP amplitude (Figure 5C, left) and MUNIX values (Figure 5C, right) at the basal visit. Figure 5D shows that the patients separated into two groups: patient numbers 10, 12, 16, and 22 with higher CMAP peak and MUNIX values and no progression (slope close to 0; Figure 5E) and patients (4, 13, 14, 18, 20) who had lower CMAP peak and MUNIX values and a clear worsening (negative slope; Figure 5E) of the CMAP peak and MUNIX. Table 4 shows the differences between these two groups of patients. We did not study the differences in the effect of the injection in these two groups due to the small number of patients. This heterogeneity is not a conclusive finding (due to the small number of patients), but it could play a role in determining the effect of tentative therapeutic strategies, such as the use of stem cells.

**TABLE 4:** Average values of several parameters in the two groups of patients detected by the cluster analysis.

	Patients: 10, 12, 16, 22	Patients: 4, 13, 14, 18, 20	*P* value: Rank sum test
CMAP amplitude (mV)	7.940.82	3.870.26	0.016
MUNIX	154.011.92	63.44.63	0.016
CMAP slope (mV/day)	0.120.58	−6.361.72	0.016
D50	41.007.52	35.802.25	Not significant
FD	2.910.41	3.520.18	Not significant

## Discussion

This clinical trial shows that the intramuscular injection of BMMCs is safe. The serious AEs detected during the follow-up were a natural consequence of the disease progression, and none were related to the procedure. This finding is consistent with previous reports of trials employing hematopoietic stem cells in ALS (Staff et al., 2016; Syková et al., 2017), including one clinical trial (Petrou et al., 2016) in which stem cells were also intramuscularly implanted. The trial also assessed efficacy: this was done by comparing the functional properties of the control and experimental TA muscles throughout the follow-up period. It is important to remark that the work was focused on a single muscle, rather than on patient survival or disease progression. Overall, we found no significant differences between the control and experimental TA muscles in parameters related to MUs number or size. The only exception was the D50 index used to quantify the CMAP scan curve, which was significantly larger in the experimental muscle than in the control muscle at the 90- and 180-day visits.

This ALS clinical trial design approach, based on comparing sides in the same patient, has recently been proposed by Rushton et al. (2017) after demonstrating that progression rate in the left and right limb muscles of ALS sufferers shows a high degree of correlation; this progression is not related to the first muscle to begin declining and, on average, the right and left sides decline at the same rate following onset. For these reasons, an approach using one side for treatment and the other as a simultaneous internal control is perfectly feasible for stem cell transplant trials.

While some of the parameters that we measured are a direct estimation of motor neuron number (statistical MUNE and MUNIX) or size (FD, MUSIX, and SMUP), the CMAP scan curve is a complex parameter related to the number and size of the MUs as well as to axonal excitability. In ALS patients, the CMAP scan curve usually shows clear morphological changes, characterized by the presence of large discrete steps. This curve may be used to estimate both the number and size of MUs in a muscle (Henderson et al., 2006); it also allows the simultaneous study of denervation and reinnervation and, eventually, muscle fiber loss (Maathius et al., 2013). The CMAP scan is especially useful for detecting reduced numbers of MUs and the presence of enlarged MUs (Visser and Blok, 2009). In addition, the CMAP scan curve is related to axonal excitability (Visser and Blok, 2009; van der Heyden et al., 2013). Of the several parameters described to quantify this curve (Henderson et al., 2007; Maathius et al., 2013; Sleutjes et al., 2014; Bostock, 2016), we have used the D50 index; this parameter decreases progressively during the progression of ALS, reflecting at least the processes of MU loss and reinnervation across a range of different disease progression rates (Sleutjes et al., 2014; de Carvalho et al., 2018). For this reason, interpreting differences between the experimental and control sides that we detected using the D50 index is complex. The larger values found in the treated sides could indicate a larger number of functional MUs and/or smaller MUs with respect to the control side. However, this was not corroborated by changes the parameters that directly estimate the number of MUs (statistical MUNE and MUNIX) or MU size (FD and MUSIX): these showed no differences between sides. The significant differences in the D50 index suggests that the BMMCs caused a specific effect in the treated muscle. This effect was only detected by this index signifying that, as a quantitative index of the CMAP scan curve, it is more sensitive than other methods (MUNE, MUNIX, and CMAP amplitude) and can detect subtle changes in MUs as well as differences in ALS progression, as recently proposed by Jacobsen et al. (2019). Also, the possible presence of muscle heterogeneities between ALS sufferers (see below) could hide the effects of injecting BMMCs. One of the inclusion criteria of this trial was a muscle strength measurement of 4 or 5 on the MRC scale, which may have resulted in a sample of patients whose TA muscles were relatively unaffected at the time of inclusion. This fact could also hide the possible effect of injecting BMMCs, as this should have no effect on healthy muscles, although it could enhance the value of the CMAP scan as it is more sensitive when studying the effect of BMMC on the functional properties of MUs. This higher sensitivity of the D50 index can support the use of this parameter to quantify the progression of MU damage and could make the CMAP scan curve a neurophysiological biomarker candidate of ALS progression, as previously suggested (Simon et al., 2014).

The effect on the D50 index may have multiple and complex mechanisms, since it involves changes in the size and/or the number of functional MUs in the TA muscle. MSCs secrete neurotrophic factors that could provide trophic support for axon terminals and neuromuscular junctions (Sadan et al., 2009); it has been shown that the intramuscular injection of bone-marrow-derived stem cells in an animal model of motor neuron degeneration causes an increase in the size of the end-plates that correlates with a greater survival of motor neurons (Pastor et al., 2013). Among different trophic factors, GDNF, which is secreted by stem cells, has been shown to promote motoneuron survival more efficiently than others (Henderson et al., 1994; Suzuki et al., 2007, 2008). In addition, MSCs have anti-inflammatory effects mediated by the secretion of cytokines (Sadan et al., 2009) and this action has been described in animal models of other neurogenerative diseases (Wei et al., 2018).

Our data clearly reveals heterogeneities among TA muscles in ALS patients. We used a hierarchical cluster analysis with five parameters: the values of the CMAP peak amplitude, MUNIX, D50, and FD from the control side at the basal visit, and the slope of the linear fit of the CMAP values measured in the control at all of the five visits making up the complete study. The slope of the linear fit of the CMAP values was used as an objective estimate of the rate of disease progression in a particular muscle, and the need to obtain this linear fit from the entire set of visits restricted this analysis to the 9 patients that completed the study and for which there was a full set of data. This analysis clearly reveals the presence of two sets of patients. The first set had higher CMAP and MUNIX values at the basal visit and the slope of the CMAP progression was close to 0; this shows that, in these patients (*n* = 4), the TA muscle was well conserved at the onset of the trial and remained intact during the follow-up period. In contrast, the second set of patients (*n* = 5) showed the opposite, indicating that in these patients, the muscle was already affected at the onset of the trial and then clearly declined during the follow-up period. Our finding of two groups of ALS patients is consistent with the classification made by others based on the evolution of CMAP values (Nandedkar et al., 2004), and the evolution of grip strength, CMAP, MUNE, and FD (Yuen and Olney, 1997). Given the low numbers of patients in the two groups, we made no further analysis of differences related to BMMC injection, but this heterogeneity may be a crucial factor for the study of therapeutic alternatives based on direct action on muscles in ALS patients.

Our work has limitations: a small number of patients and the heterogeneity of the TA muscle damage. The number of patients was determined by the trial protocol and it is adequate for Phase I/II trials whose primary objective is to determine the safety of the procedure in ALS patients; also, at the time of designing the protocol, we did not have previous data about intramuscular stem cell effects in MU properties. The heterogeneity of patients was an additional source of variability that could mask the effects of intramuscular stem cells.

## Conclusion

We evidence the safety of intramuscular injection of BMMCs into ALS patients. The difference found in the D50 index strongly suggests that the BMMCs may be acting on the number and/or size of the MUs in the TA muscle and that this parameter may be especially sensitive for studying the effects of tentative treatments acting on MUs. These results, together with the finding of muscle heterogeneity in ALS suffers, justify further analysis in the form of Phase II trials involving both a larger patient sample and other muscles.

## Data Availability Statement

The datasets generated for this study are available on request to the corresponding author.

## Ethics Statement

The studies involving human participants were reviewed and approved by Ethics committees of the Hospital Virgen de la Arrixaca and the Hospital Universitario de San Juan. The trial protocol was approved by the Agencia Española del Medicamento of the Spanish Ministry of Health. The patients/participants provided their written informed consent to participate in this study.

## Author Contributions

EG-B, CP-O, and PM designed the study, acquired and analyzed the data, and drafted the manuscript for intellectual content. MB designed the study, acquired and analyzed the data, and revised the manuscript for intellectual content. JE, NI, and AG-H acquired the data. FI, JM, and SM designed the study, interpreted the data, and revised the manuscript for intellectual content. CM-E designed the study. LB interpreted the data. All authors approved the manuscript for publication.

## Conflict of Interest

The authors declare that the research was conducted in the absence of any commercial or financial relationships that could be construed as a potential conflict of interest.
